# Definition and Assessment of Paediatric Breakthrough Pain: A Qualitative Interview Study

**DOI:** 10.3390/children11040485

**Published:** 2024-04-18

**Authors:** Eleanor Dawson, Katie Greenfield, Bernie Carter, Simon Bailey, Anna-Karenia Anderson, Dilini Rajapakse, Kate Renton, Christine Mott, Richard Hain, Emily Harrop, Margaret Johnson, Christina Liossi

**Affiliations:** 1 School of Psychology, University of Southampton, Highfield SO17 1BJ, UKkatiemgreenfield@gmail.com (K.G.); 2 Faculty of Health, Social Care and Medicine, Edge Hill University, St Helens Road, Ormskirk L39 4QP, UK; bernie.carter@edgehill.ac.uk; 3 Department of Children’s Oncology, Great North Children’s Hospital, Queen Victoria Road, Newcastle upon Tyne NE1 4LP, UK; 4 Royal Marsden Hospital, Downs Road, Sutton SM2 5PT, UK; annakarenia.anderson@nhs.net; 5 The Louis Dundas Centre, Hospital for Children NHS Foundation Trust, Great Ormond Street, London WC1N 3JH, UK; 6 University Hospital Southampton NHS Trust, Tremona Road, Southampton SO16 6YD, UK; 7 Naomi House & Jacksplace, Stockbridge Road, Sutton Scotney, Winchester SO21 3JE, UK; 8 Acorns Children’s Hospice, 103 Oak Tree Lane, Selly Oak, Birmingham B29 6HZ, UK; 9 Birmingham Children’s Hospital, Birmingham, Steelhouse Lane, Birmingham B4 6NH, UK; 10 Department of Child Health, Swansea University, Swansea SA2 8PP, UK; richard.hain@wales.nhs.uk; 11 Helen & Douglas House Hospices, 14A Magdalen Road, Oxford OX4 1RW, UK; eharrop@helenanddouglas.org.uk; 12 Oxford University Hospitals NHS Trust, John Radcliffe Hospital, Headley Way, Headington, Oxford OX3 9DU, UK; 13 Patient & Public Representative, Oxford OX4 1RW, UK; ottersriverbank@natsirt.co.uk; 14 Psychological Medicine, Hospital for Children NHS Foundation Trust, Great Ormond Street, London WC1N 3JH, UK

**Keywords:** breakthrough pain, pediatrics, palliative care, pain measurement, interview, delivery of healthcare

## Abstract

Infants, children and young people with life-limiting or life-threatening conditions often experience acute, transient pain episodes known as breakthrough pain. There is currently no established way to assess breakthrough pain in paediatric palliative care. Anecdotal evidence suggests that it is frequently underdiagnosed and undertreated, resulting in reduced quality of life. The development of a standardised paediatric breakthrough pain assessment, based on healthcare professionals’ insights, could improve patient outcomes. This study aimed to explore how healthcare professionals define and assess breakthrough pain in paediatric palliative care and their attitudes towards a validated paediatric breakthrough pain assessment. This was a descriptive qualitative interview study. Semi-structured interviews were conducted with 29 healthcare professionals working in paediatric palliative care across the UK. An inductive thematic analysis was conducted on the data. Five themes were generated: ‘the elusive nature of breakthrough pain’, ‘breakthrough pain assessment’, ‘positive attitudes towards’, ‘reservations towards’ and ‘features to include in’ a paediatric breakthrough pain assessment. The definition and assessment of breakthrough pain is inconsistent in paediatric palliative care. There is a clear need for a validated assessment questionnaire to improve assessment, diagnosis and management of breakthrough pain followed by increased healthcare professional education on the concept.

## 1. Introduction

It is estimated that over 86,000 infants, children and young people in England are living with life-limiting or life-threatening conditions [1]. The definition of breakthrough pain (BTP) varies [2] but is broadly seen as a temporary increase in pain severity over and above background (or baseline) pain (mild-moderate pain for ≥12 h/day [3]). It is common in children with cancer [4], life-limiting conditions [5] and at end-of-life [6]. Paediatric BTP may be underdiagnosed [4] and undertreated [5,7], causing reduced wellbeing [8,9,10].

Despite considerable efforts, experts have yet to agree on a universal BTP definition [2,11]. Table 1 displays example definitions from a comprehensive literature review. These are largely intended for adult cancer populations and the authors do not state whether they apply to children. Several describe BTP as occurring in patients with background pain controlled by opioids. However, clinicians may be reluctant to prescribe opioids to younger children [12]. Moreover, there is no consensus on what constitutes ‘stable’ background pain. Some experts believe BTP can occur irrespective of adequate background pain control, and even without background pain [13,14]. Thus, these definitions could lead to underdiagnosis and inadequate treatment of paediatric BTP and considerable distress for families.

**Figure 1:**
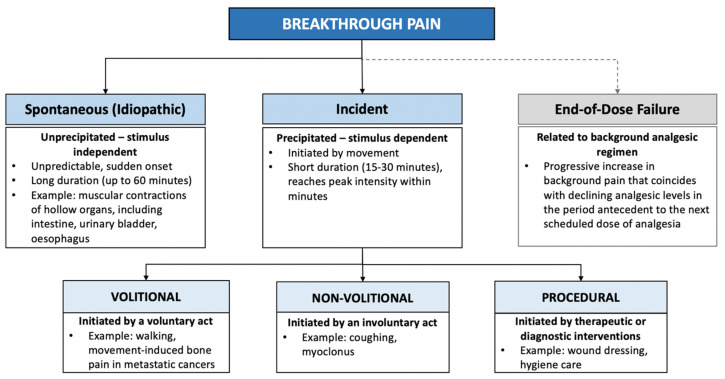
Schematic diagram of potential subtypes of breakthrough pain [3,27,28]. Note: bold lines indicate subtypes that are commonly cited in the literature; dotted lines indicate subtypes that are generally not considered to be breakthrough pain.

BTP is complex (see Figure 1) and can include subtypes such as incident or spontaneous pain [3]. End-of-dose failure has been suggested as a third subtype [19], though its pharmacokinetic profile suggests it is more reflective of poorly controlled background pain [7,20]. BTP can have neuropathic, visceral, somatic, or mixed pathophysiologic mechanisms [7]. These can align with, or differ from, background pain mechanisms, which can complicate pain management if not appropriately assessed [16,21]. In the adult literature, BTP is typically described as moderate-severe intensity [22,23], of rapid onset [23,24], and lasting between 15–30 min [25]. Far less is known about paediatric BTP characteristics, though limited evidence suggests that it is sudden and can occur multiple times a day [4], lasting from seconds to minutes [26].

Paediatric palliative care covers neonates to teenagers, many of whom have impaired social, emotional, cognitive and neurological function [29] and varied diagnoses [4], affecting their capacity to self-report pain [30,31]. Experts recommend validated assessment questionnaires to diagnose and manage BTP [7,32,33]. However, of the 13 BTP questionnaires identified by a recent systematic review, only one—The Breakthrough Pain Questionnaire for Children [34]—was designed for children [35,36]. It is only suitable for 7–18-year-olds who can self-report. There are no published studies evaluating its measurement properties [36].

A recent review of children’s clinical records at end-of-life [5] identified stark inconsistencies in BTP reporting by healthcare professionals (HCP). Significant variation in characteristics and terminology used to diagnose BTP can limit standardisation and quality of care [5,37]. To understand how healthcare professionals conceptualize, define and assess paediatric breakthrough pain in their everyday clinical practice, qualitative methods were used. These methods allow for a deep, contextual understanding of real-world issues in healthcare, including people’s beliefs, perspectives, and lived experiences, as well as the challenges they face [38]. Such a detailed understanding is currently missing from the literature regarding paediatric BTP. The present study explored how HCP define and assess BTP in paediatric palliative care and their attitudes towards a validated paediatric BTP pain assessment.

## 2. Materials and Methods

This descriptive qualitative interview study was part of the Paediatric Palliative Care Pain Management in the Community (PARAMOUNT) study, a multicentre study aiming to improve end-of-life paediatric pain in the community. National and local ethics approvals were obtained [London−Bloomsbury Research Ethics Committee (IRAS reference: 262102)] on 2 August 2019. There are many qualitative methods used in health services research including ethnography, grounded theory, framework analysis, content analysis, reflexive thematic analysis and others [38]. We chose thematic analysis due to its flexibility. This method enables in-depth exploration of participants’ personal views and the identification of thematic patterns within data, proving particularly useful when handling large data sets [39]. Standards for Reporting Qualitative Research (SRQR) guidelines are followed [40].

A purposive sample of HCPs working in the UK and caring for 0–18-year-olds at end-of-life across primary, secondary, and tertiary care was recruited. ‘End-of-life’ was defined using the Spectrum of Palliative Care Needs classification framework (orange or red categories [41]) as death that would not be unexpected within the next five years. The study was advertised using posters, email lists, websites and social media. Posters were displayed at participating clinical centers, and the study was advertised on the University of Southampton’s website. Additionally, the study was promoted on LinkedIn and in Twitter (now X). Email lists of healthcare professionals working in paediatric palliative care or pain were utilized, e.g., Pediatric-Pain List, the Children, Young People Nurse Academics UK (CYPNAUK) list. Recruitment continued until data saturation [42].

Semi-structured, open-ended interviews were conducted. An interview guide was co-produced by the team with M.J., a parent of a child who had palliative needs. M.J. provided specific real-life insights to the lives of families and helped frame the wording of questions. The guide was also informed by a systematic review of paediatric symptom management at end-of-life [43,44]. This included questions about paediatric palliative pain assessment and management, how participants defined breakthrough pain, and their thoughts on a paediatric BTP assessment. Findings from the broader questions have been published [45]; this article reports findings from the questions relating to BTP only.

Interviews were conducted by K.G, S.H, and E.D between September 2019 and March 2020 in hospitals, hospices, and primary care centres. One group (n = 3) telephone interview was conducted due to COVID-19 pandemic restrictions. All participants provided informed written consent.

Data analysis was undertaken by a team consisting of academics and clinicians (female and male) representing medicine, nursing and psychology with experience of caring for children with breakthrough pain in a variety of settings (see Table 2 for data analysis). Our analysis was reviewed by our parent representative who provided feedback which informed the final themes.

The four criteria for trustworthiness [47] were paramount throughout the analysis process; for example, credibility was ensured by triangulating codes and perspectives across the team, we have provided detailed description assuring transferability, we ensured dependability through a clear audit trail of decision-making and the entire process involved high levels of reflexivity creating confirmability.

## 3. Results

**Figure 2:**
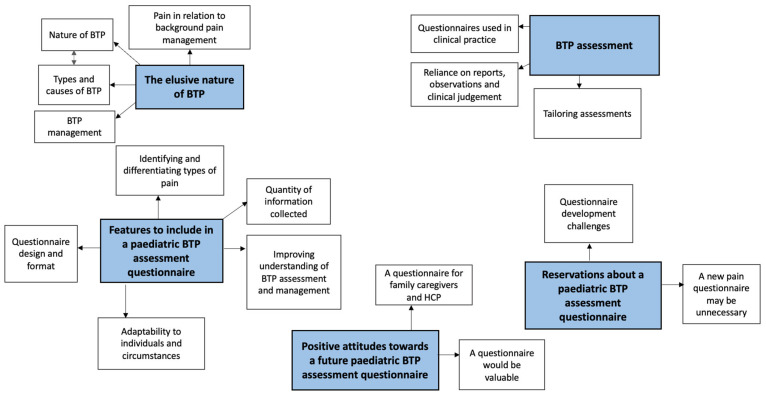
Themes generated from a thematic analysis of a qualitative interview study exploring healthcare professionals’ views on the definition of paediatric breakthrough pain and the development of a validated breakthrough pain assessment tool for use in paediatric palliative care (n = 29). Key: BTP—breakthrough pain, HCP—healthcare professionals. Blue boxes—themes; solid lines—subthemes.

We conducted 27 interviews with 29 HCPs (see Table 3). Five overarching themes were generated: ‘the elusive nature of BTP’, ‘BTP assessment’, ‘positive attitudes towards’, ‘reservations towards’ and ‘features to include in’ a paediatric BTP pain assessment (see Figure 2). Findings were broadly similar between subgroups of professionals; major differences are reported in the text.

### 3.1. The Elusive Nature of BTP

Defining BTP was challenging. Its nature was elusive to clear description and was often presented in relation to, and in the context of, other factors, explored below.

#### 3.1.1. Pain in Relation to Background Pain Management

BTP was widely defined as any type of pain occurring in addition to, or despite, a background pain management regimen; ‘breakthrough pain is any pain that is literally breaking through despite the background pain management’ (P17, GP-hospice). Most considered BTP to be a consequence of inadequately controlled background pain requiring additional analgesia; ‘the breakthrough pain can…be that the baseline pain is not adequately managed’ (P10, Doctor-hospital). Only one participant defined BTP as occurring irrespective of background pain regimens.

#### 3.1.2. Nature of BTP

BTP could be predictable (e.g., due to a build-up of brain tumour-related pressure causing headaches) or unpredictable with no obvious trigger. Episodes were described as acute, severe, intermittent and of sudden onset, ‘it’s not there all the time, it’s intermittent and there are peaks of pain’ (P11, Doctor-hospital); and ‘they will suddenly get a burst of pain’ (P19, Nurse-hospice).

Despite being distinct from pain, the words ‘distress’ or ‘discomfort’ were commonly used in BTP descriptions by those with more paediatric palliative care experience. One participant proposed that BTP could be ‘pain or distress or discomfort which might be physical or psychological’ (P24, Support therapist-hospital). Another described it as ‘when somebody becomes so uncomfortable that…they need to have a breakthrough dose of medication’ (P20, Nurse-hospice).

#### 3.1.3. Types and Causes of BTP

Participants referred to various types and causes of BTP. A commonly cited cause was movement due to volitional, non-volitional, and procedural actions (e.g., coughing, nappy changes, postural repositioning). More experienced participants made a clear distinction between spontaneous (unpredictable and occurring without an obvious trigger), and ‘incident pain’ (predictable and often movement-induced). A consultant explained ‘breakthrough is what happens at rest and incident pain is maybe what happens when…that sense of movement and change brings about the pain. So, I think the two are a bit different’ (P3, Doctor-hospice). BTP was seen as distinct from end-of-dose failure.

#### 3.1.4. BTP Management

BTP is complex and ‘very difficult maybe to manage’ (P16, GP-hospice). Definitions focused on management strategies, which commonly involved fast-acting analgesia. Management was pre-emptive ‘before changing dressings or before moving beds’ (P16, GP-hospice) or reactive ‘they then get like a surge of pain and then they need a breakthrough dose of medication to make that then stable again’ (P20, Nurse-hospice). When children experience several episodes of BTP in a day, participants noted that their background pain management was inadequate and required adjustment. Non-pharmacological interventions were proposed ‘sometimes a bit of distraction or a bit of massage…and then they won’t need the breakthrough medication’ (P20, Nurse-hospice) by more experienced participants.

### 3.2. BTP Assessment

Nurses and doctors used a holistic approach to BTP assessment. This involved caregiver’ reports, questionnaires, symptom management plans, and HCP’s clinical experience and knowledge of the child.

#### 3.2.1. Questionnaires Used in Clinical Practice

No participants reported using a BTP-specific assessment. Several noted that one did not exist in paediatrics. Instead, most used unidimensional pain assessments (e.g., numeric rating and visual analogue scales, Faces Rating Scales [48], and Face Legs Activity Cry Consolability (FLACC) scale [49]. These were designed to identify the presence and intensity of pain but not its clinical features (e.g., duration, cause and context). Frequency of assessment use varied from hourly, ‘we just plot it on a graph, so we’d have like an hourly graph’ (P20, Nurse-hospice) to as required, ‘it’s not like we just assess it on the hour every hour…we do it as required, say if the child has said they’re in pain’ (P19, Nurse-hospice).

#### 3.2.2. Reliance on Reports, Observations, and Clinical Judgement

Many participants relied on ‘gut instinct’ and clinical experience to diagnose BTP. Where children were able, self-report was highly valued as children were viewed as experts of their own pain experience. Parent and colleague reports were also useful, ‘you really need to rely on the parents to…tell you and sort of make a plan together to manage’ (P24, Support therapist-hospital). Collaboration between families and HCPs was critical as ‘we tend to use an agreed set of observations for each child, which we then would put in a symptom management plan’ (P3, Doctor-hospice). Compared to a single pain assessment score, these observations provided rich, multidimensional information.

#### 3.2.3. Tailoring Assessments

The importance of tailoring assessments depending on age, condition, and developmental abilities was emphasised, ‘everything’s very individual, it’s a, a totally personalised care’ (P14, GP-community) and ‘whether we use the personalised pain assessment chart or whether we use…numbers, faces, whatever is appropriate for that young person’ (P19, Nurse-hospice). A doctor explained that ‘rather than use specifically a FLACC…we’re more likely to say something about that child, because so many of my children are not…able to communicate, it’s very difficult… some of them do very strange things like laugh when they’re in pain’ (P3, Doctor-hospice). Contextual information and potential causes also formed an important part of assessment.

### 3.3. A Validated Questionnaire Is Needed-Positive Attitudes

#### 3.3.1. A Questionnaire Would Be Valuable

Overall, participants, particularly those in community and hospital settings, and with >11 years’ experience working in paediatric palliative care, were receptive towards a paediatric BTP assessment questionnaire. A pharmacist explained that ‘we definitely need…more objective assessment questionnaires and it would aid analysis of knowing which strategies are effective for what pain’ (P8, Pharmacist-hospital). Participants felt that a questionnaire that could characterise and distinguish breakthrough pain from other pain types would be helpful. Many gave recommendations for how this could be employed. Some noted that users should not over-rely on an assessment questionnaire but instead use it alongside other information such as symptom management plans.

#### 3.3.2. A Questionnaire for Family Caregivers and HCPs

A questionnaire that could be used by parents and HCPs would be valuable; ‘I’m all for anything…that, that can help me to assess a child suitably, and if parents can use it as well, even better’ (P2, Nurse-community). ‘A lot of parents are worried about overdosing their child so actually…it [a questionnaire] might give them reassurance that actually when their child does need the pain relief it’s ok to give it’ (P25, Doctor-community).

### 3.4. Reservations towards a Paediatric BTP Assessment Questionnaire

Some participants expressed reservations about the development and necessity of a questionnaire.

#### 3.4.1. Questionnaire Development Challenges

Some participants queried how such a complex construct could be measured; ‘do you use the same sort of visual scale?…it’s how you practically assess it and measure it and log it’ (P017, GP-hospice). Another noted that validating a questionnaire ‘for something that has not been truly defined would be tricky’ (P13, Nurse-hospital). A hospital nurse mentioned the challenge of recruiting participants to validate the questionnaire, particularly those who were not neurotypical.

#### 3.4.2. A New Pain Assessment Questionnaire May Be Unnecessary

A few participants questioned the need for a questionnaire to assess BTP separately from other pain types as ‘pain is pain whether it’s breakthrough or not so could you not use the same scales?’ (P2, Community nurse). A nurse queried how the questionnaire would work with ‘a regular pain assessment questionnaire’ as ‘sometimes it’s simpler just to use one thing unless there’s a very good way that they kind of interrelate’ (P19, Nurse-hospice).

### 3.5. Features to Include in an Assessment

Participants talked of a range of different features that should be included in a questionnaire.

#### 3.5.1. Identifying and Differentiating Pain Types

Participants wanted the questionnaire to assess onset, cause, pathophysiology, and temporality as ‘it would be helpful to know more about it, more about what type of pain, whether it’s more to do with aching or neuropathic [pain]…that kind of thing’ (P5, Nurse-community). The questionnaire should also ‘help distinguish between breakthrough pain, incident pain, and end-dose failure pain’ (P11, Doctor-hospital).

#### 3.5.2. Improving Understanding of Assessment and Management

The questionnaire should be suitable for caregivers, HCP, and patients where possible. The need for caregiver education on BTP was emphasised as ‘we don’t give them [parents] competency-based training in pain assessment, and should we?…We send them home to manage some of the [most] complex pain [in] the world, we don’t actually verify them as competent to do that’ (P3, Doctor-hospice).

More BTP education for HCPs was suggested as ‘sometimes HCPs will say…’they’re [the child is] still in pain, I can’t give anything else’ and you’ll say, ‘well have you given any breakthrough?’ and they’ll say, ‘no because they’ve just had their long acting [pain medication]’’ (P9, Nurse-community). Improved pain management, communication, and teamwork between families and clinicians was anticipated if both parties used the same assessment, by ‘help[ing] to join up the dots a bit and connect up, make it clearer’ (P26, Support therapist-hospital).

#### 3.5.3. Adaptability to Individuals and Circumstances

The questionnaire must ‘be open enough so they can fit it to their patient and their situation’ (P3, Doctor-hospice) since circumstances and pain signs are unique, and there is a wide developmental and cognitive range in paediatric palliative care patients. One proposition to achieve this was the ‘idea of a language or a currency per child’ (P3, Doctor-hospice doctor) to describe their individual BTP signs, which could be used alongside a standardised assessment questionnaire.

#### 3.5.4. Quantity of Information Collected

Participants working in hospital and hospice settings were hesitant about relying exclusively on a simple pain scale that did not incorporate caregiver and child pain reports since ‘you have to do a history taking, you’ve really got to explore that pain experience and understand it from every angle you can’ (P13, Nurse-hospital). However, concern was expressed about a very detailed, lengthy questionnaire that could cause unnecessary caregiver burden as ‘what they [parents] don’t like is collecting endless data afterwards, even in good times, because it just breaks them’ (P3, Doctor-hospice).

#### 3.5.5. Questionnaire Design and Format

A standardised way to record key pain features and treatment was considered helpful as this could help distinguish BTP from other pain types and identify triggers, thus provide more effective pain management. Using a visual format (e.g., a graph) was expected to help ‘see what works, what doesn’t’ (P20, Nurse-hospice) and when further medications are needed. It was suggested that the questionnaire could a smartphone application (app), which both family caregivers and HCPs could easily use, access and share.

## 4. Discussion

To our knowledge, this is the first study to investigate how paediatric palliative care professionals define and assess BTP, and to explore their views on a paediatric BTP assessment. A key finding was a lack of consensus on the definition of paediatric BTP. Our findings indicate a clear need for a BTP assessment and provide valuable insights into, and suggestions, for the best type, design, and content, which will help its development and validation [50].

Many themes were based on data from nurses and doctors rather than pharmacists and support therapists, which could be because the interview was focused on pain assessment. Questions about pain management may have provided data more reflective of the entire care team. Another limitation of the study is that, due to our recruitment strategy, we cannot ascertain who we reached or the representativeness of our sample among healthcare professionals caring for children and young people experiencing breakthrough pain.

Adult oncology research indicates that clinicians who described BTP as occurring in the context of controlled background pain prepared more effective BTP management strategies than those who felt it occurred regardless of background pain [51]. This suggests that the way HCP define BTP has direct implications on patient outcomes. In the current study, definitions of BTP varied, which is unsurprising given the lack of a universally accepted definition [14] and the dearth of research in paediatric BTP. While some described it as occurring in the context of controlled background pain, others felt it was indistinguishable from poorly controlled background pain. These differing definitions may affect pain management choices and outcomes, as they do in the adult oncology literature. These findings strongly suggest the need for more BTP education following a consensus on the BTP definition.

Some participants reported that BTP could be triggered by specific events, which broadly corresponds with the adult literature [20]. However, some of the more experienced professionals considered it was distinct from incident pain. In keeping with a recent consensus statement [3], BTP was not seen as equivalent to end-of-dose failure. Managing the emotional effects of BTP through non-pharmacological strategies was mentioned by several participants, indicating a holistic approach to pain management. This is in line with the World Health Organisation’s recommendations to use both pharmacological and non-pharmacological interventions to manage paediatric pain at end-of-life [52]. It also reflects the recently updated definition of pain by the International Association for the Study of Pain as “an unpleasant sensory and emotional experience associated with, or resembling that associated with, actual or potential tissue damage” [53].

Our findings correspond with results from an observational study indicating a lack of consistency in assessing and documenting BTP in paediatric palliative care [5]. The study reported that methods for assessing BTP varied and included caregiver and patient reports, observations from colleagues, clinical experience, and generic pain assessment questionnaires. Participants in the current study noted that some families found it too burdensome to regularly assess and record pain. A lack of uniformity in BTP assessment can negatively impact pain management [5] and consequently children’s and parents’ physical and psychological wellbeing [9,54]. As such, our findings further highlight the need for a validated assessment questionnaire.

No participants in our study used a BTP-specific assessment questionnaire or were aware of such a questionnaire for children, though generic questionnaires such as a visual analogue scale were used. However, these are limited since they cannot discriminate background from BTP [55] and can lead to underdiagnosis and undertreatment of BTP [56]. More experienced participants in our study conducted more thorough, holistic assessments incorporating information from multiple sources, including parent reports, which were viewed as invaluable for collecting detailed pain information. They acknowledged the need for assessments that were tailored to children’s developmental stage and cognitive abilities, in line with the National Institute for Health and Care Excellence (NICE) guidance for paediatric end-of-life care in infants, children and young people [57].

Currently no validated paediatric BTP assessment questionnaire exists [36] yet most participants felt a robust questionnaire would be valuable. This is an important finding since positive attitudes towards standardised assessment questionnaires are associated with increased use [58].

Participants proposed that the questionnaire should not be too time-consuming since it is important to manage BTP quickly and minimise burdensome tasks for families [59]. However, they wanted it to go beyond a simple pain scale and provided information about clinical pain features (e.g., temporality, cause, pathophysiology). They also valued patient, caregiver, and colleague reports. As such, the questionnaire would ideally have self-report and caregiver-report versions, particularly since parental involvement in symptom management at end-of-life is associated with increased parental satisfaction with care [60].

The questionnaire would need to be suitable for children across a range of ages, abilities and conditions, including those with severe disabilities who may have atypical pain signs [61]. It could be designed so that families and professionals produce an agreed set of pain signs that could be incorporated into the assessment questionnaire.

Standardised assessment and recording of BTP could aid teamwork and communication between families and professionals, and within clinical teams [5]. It may also help to identify pain triggers and patterns [36]. An electronic questionnaire, perhaps a smartphone app, could be beneficial since information could be stored in one place and shared easily. This aligns with other findings showing that electronic pain diaries were associated with significantly higher adherence and accuracy than paper pain diaries in children with chronic pain [62]. Apps for assessing and recording paediatric pain have been positively reviewed by patients, including children with cancer, parents and HCP, and are associated with higher adherence rates compared to paper-based versions [63,64]. Digital pain reports could also be more easily added to patients’ digital health records.

## 5. Conclusions

Inadequate assessment and a lack of validated pain questionnaires are significant barriers to adult BTP diagnosis and management [31,65]. Poorly managed BTP detrimentally affects patients and caregivers’ quality of life [66,67]. Research in paediatric BTP is lacking. Our findings indicates that definitions and assessment are inconsistent; this could prevent appropriate diagnosis and management. A consensus on the definition of paediatric BTP and subsequent education for HCP is needed. There is a clear requirement for a validated paediatric BTP questionnaire that is developed with, and can be used by, families and HCP to improve BTP diagnosis and management.

## Figures and Tables

**Table 1:** Example breakthrough pain definitions in published literature.

Definition
Portenoy and Hagen, 1989 [15]: ‘a transitory increase in pain to greater than moderate intensity which occurs on a baseline of pain of moderate intensity or less’ (p. 25)
Portenoy and Hagen, 1990 [16]: ‘a transitory exacerbation of pain that occurs on a background of otherwise stable pain in a patient receiving chronic opioid therapy’ (p. 273)
Davies, Dickman et al., 2009 [3]: ‘a transient exacerbation of pain that occurs either spontaneously, or in relation to a specific predictable or unpredictable trigger, despite relatively stable and adequately controlled background pain’ (p. 332)
European Association of Palliative Care, 2012 [17]: ‘transitory exacerbations of pain that occur on a background of stable pain otherwise adequately controlled by around-the-clock opioid therapy’ (p. e62)
World Health Organisation, 2012 [18]: ‘a temporary increase in the severity of pain over and above the pre-existing baseline pain level’ (p. 8)

**Table 2:** Process used for data analysis of interviews with healthcare professionals exploring their views on the definition of paediatric breakthrough pain and the development of a validated breakthrough pain assessment questionnaire for use in paediatric palliative care.

Interviews were audio-recorded and transcribed verbatim, with all identifying information removed. Transcripts were imported into NVivo (Version 12) [46]
2. A contextualist epistemology and critical realist ontology were assumed
3. E.D. and K.G. conducted an inductive thematic analysis [39] to identify patterns within the data. Data analysis followed the six-phase strategy outlined by Braun and Clarke [39]
4. Transcripts were read several times prior to coding to ensure data immersion
5. Line-by-line coding was used to identify recurring features across interviews
6. Codes were then collated into candidate themes and used to develop a coding manual which was applied across the entire data set. The coding manual was iteratively developed by E.D., K.G., B.C., and C.L. to ensure it clearly represented the data. Coding was conducted independently by E.D. and K.G.; discrepancies were discussed and resolved with B.C., and C.L.
7. Subgroup analyses were conducted to explore differences in findings across profession (nurse, general practitioner, consultant and registrar doctor, pharmacist, and support therapist), work setting (hospice, hospital, and community setting), and years’ experience (<1, 1–4, 5–10, 11–15, and >15 years)

**Table 3:** Demographics of healthcare professionals who participated in interviews exploring their views on the definition of paediatric breakthrough pain and the development of a validated breakthrough pain assessment questionnaire for use in paediatric palliative care (n = 29).

Demographics	
Age (average ± SD)	44.6 ± 8.1 years
Age range	26–61 years
Female	n = 25 (86.2%)
Role	
Nurse	n = 12 (41.4%)
GP	n = 5 (17.2%)
Consultants and registrar doctors	n = 5 (17.2%)
Pharmacist	n = 2 (6.9%)
Psychological, social & physical support therapists	n = 5 (17.2%)
Work setting	
Community	n = 9 (31.0%)
Hospice	n = 10 (34.5%)
Hospital	n = 10 (34.5%)
Years in paediatric palliative care (average ± SD)	11.1 ± 8.1 years
Years in paediatric palliative care (range)	2 months–25 years

Note: To protect participants’ anonymity, consultants and registrar doctors were grouped into one category and psychologists, counsellors, play therapists, occupational therapists and social workers were grouped into another. Participants working across more than one setting were allocated the setting in which most of their work was conducted.

## Data Availability

Anonymised qualitative data will be stored in a restricted cloud-based University system (SharePoint) and will be shared with third parties only upon reasonable request.
